# Behavior of tricellulin during destruction and formation of tight junctions under various extracellular calcium conditions

**DOI:** 10.1007/s00441-012-1512-7

**Published:** 2012-10-17

**Authors:** Akira Takasawa, Takashi Kojima, Takafumi Ninomiya, Mitsuhiro Tsujiwaki, Masaki Murata, Satoshi Tanaka, Norimasa Sawada

**Affiliations:** 1 Department of Pathology, Sapporo Medical University School of Medicine, S1, W17, Sapporo, 060-8556 Japan; 2 Department of Anatomy, Sapporo Medical University School of Medicine, Sapporo, Japan

**Keywords:** Tight junction, Tricellulin, Phosphorylation, Extracellular calcium, Barrier, Fence, Human pancreatic cancer cell line

## Abstract

Tricellulin is an important component of tricellular tight junctions (TJs) and is involved in the formation of tricellular contacts. However, little is known about its regulation during the assembly and disassembly of tricellular TJs. By using the well-differentiated pancreatic cancer cell line HPAC, which highly expresses tricellulin at tricellular contacts, we have investigated changes in the localization, expression and phosphorylation of tricellulin and in its TJ functions as a barrier and fence during the destruction and formation of TJs induced by changes in the extracellular calcium concentration. During both extracellular Ca^2+^ depletion caused by EGTA treatment and Ca^2+^ repletion after Ca^2+^ starvation, the expression of tricellulin increased in whole lysates and in Triton-X-100-insoluble fractions without any change in its mRNA. The increases in immunoreactivity revealed by Western blotting were prevented by alkaline phosphatase treatment. Immunoprecipitation assays showed that tricellulin was phosphorylated on threonine residues when it increased after Ca^2+^ depletion and repletion. In the early stage after Ca^2+^ repletion, tricellulin was expressed not only at tricellular contacts but also in the cytoplasm and at bicellular borders. In confocal laser microscopy, tricellulin was observed at the apical-most regions and basolateral membranes of tricellular contacts after Ca^2+^ repletion. Knockdown of tricellulin delayed the recovery of the barrier and fence functions after Ca^2+^ repletion. Thus, the dynamic behavior of tricellulin during the destruction and formation of TJs under various extracellular calcium conditions seems to be closely associated with the barrier and fence functions of TJs.

**Electronic supplementary material:**

The online version of this article (doi:10.1007/s00441-012-1512-7) contains supplementary material, which is available to authorized users.

## Introduction

Tight junctions are the most apical components of intercellular junctional complexes. They inhibit solute and water flow through the paracellular space (termed the “barrier” function; Schneeberger and Lynch 1992; Gumbiner 1993). They also separate the apical from the basolateral cell surface domains, thereby establishing cell polarity (termed the “fence” function; van Meer and Simons 1986; Cereijido et al. 1998). The tight junction is formed not only by the integral membrane proteins such as the claudins, occludin and junctional adhesion molecules (JAMs) but also by many peripheral membrane proteins (Tsukita et al. 2001; Sawada et al. 2003; Schneeberger and Lynch 2004) that are regulated via distinct signal transduction pathways (González-Mariscal et al. 2008; Kojima et al. 2009).

The tricellular tight junction forms at the convergence of bicellular tight junctions where three epithelial cells meet in polarized epithelia and is required for the maintenance of the transepithelial barrier (Ikenouchi et al. 2005). Recently, tricellulin has been identified as the third integral tight junction protein that predominantly localizes at tricellular tight junctions. Because of this, tricellulin has been characterized as the first marker of the tricellular tight junction in epithelial cells and its loss affects the organization of the tricellular tight junction and the barrier function of epithelial cells (Ikenouchi et al. 2005). Tricellulin is highly expressed in normal human pancreatic ducts and in well-differentiated ductal adenocarcinomas (Yamaguchi et al. 2010; Korompay et al. 2012). Furthermore, its mRNA expression has been detected in the human monocytic cell line THP-1 and in mature dendritic cells (Ogasawara et al. 2009). Similar to occludin and claudins, tricellulin contains four transmembrane domains with N- and C- terminal cytoplasmic tails, the C-terminal tail exhibiting homology to the occludin C-terminus. Both tricellulin and occludin contain the tetra-spanning MARVEL (MAL and related proteins for vesicle trafficking and membrane link) domain that is present in proteins involved in membrane apposition and is concentrated in cholesterol-rich microdomains (Sánchez-Pulido et al. 2002). More recently, the lipolysis-stimulated lipoprotein receptor (LSR), originally identified and analyzed as a receptor for the uptake of triacylglyceride-rich lipoproteins (Yen et al. 1999), has been defined as an integral membrane protein localized at tricellular tight junctions and the cytoplasmic domain of LSR has been reported to be responsible for the recruitment of tricellulin (Masuda et al. 2011).

Knockdown or overexpression of occludin causes the mislocalization of tricellulin to bicellular tight junctions (Ikenouchi et al. 2008). Tricellulin is diminished by LSR knockdown, whereas LSR still accumulates at tricellular contacts after tricellulin knockdown (Masuda et al. 2011). The repression of tricellulin expression is related to Snail-induced epithelial mesenchymal transition in human gastric carcinoma (Masuda et al. 2010). We have recently reported that c-Jun N-terminal kinase (JNK) is involved in the regulation of tricellulin expression and prevents the disruption of the epithelial barrier in inflammation in human pancreatic duct epithelial cells (Kojima et al. 2010). However, little is known about the regulation of tricellulin during the assembly and disassembly of tight junctions.

The phosphorylation of tight junction proteins appears to play a central role in the regulation of tight junction assembly and function. In particular, multiple kinases targeting occludin and thereby modulating tight junction assembly, structure and function have been characterized and specific serine, threonine and tyrosine residues targeted by these kinases have been identified (Dörfel and Huber 2012). Like occludin, tricellulin appears to be phosphorylated (Ikenouchi et al. 2005). However, the phosphorylation of tricellulin has not been studied in detail.

In this study of the polarized human pancreatic cancer cell line HPAC, in which tricellulin is highly induced at tricellular contacts, we have examined the behavior of tricellulin during the destruction and formation of tight junctions in response to changes in extracellular calcium concentrations. Dynamic changes in the distribution and phosphorylation of tricellulin have been observed during the destruction and formation of tight junctions.

## Materials and methods

### Antibodies, activators and inhibitors

Rabbit polyclonal anti-tricellulin (c-term), anti-occludin, anti-JAM-A, anti-claudin-1, anti-claudin-7 and anti-phosphothreonine and mouse monoclonal anti-occludin (OC-3F10), anti-claudin-1 (2H10D10) and anti-claudin-4 (3E2C1) antibodies were obtained from Zymed Laboratories (San Francisco, Calif., USA). Rabbit polyclonal anti-actin and mouse monoclonal anti-E-cadherin antibodies were obtained from Sigma-Aldrich (St Louis, Mo., USA). Alexa 488 (green)-conjugated anti-rabbit IgG and Alexa 594 (red)-conjugated anti-mouse IgG antibodies were purchased from Molecular Probes (Eugene, Ore., USA) and anti-rabbit IgG conjugated to 12-nm colloidal gold was purchased from Jackson ImmunoResearch Laboratories (Western Grove, Pa., USA).

### Culture of cell line and treatments

Human pancreatic cancer cell line HPAC was purchased from the America Type Culture Collection (Manassas, Va., USA). HPAC cells were maintained with DMEM (Sigma-Aldrich) supplemented with 10% dialyzed fetal bovine serum (FBS; Invitrogen, Carlsbad, Calif., USA). The medium for the cell line contained 100 U/ml penicillin and 100 μg/ml streptomycin and all cells were plated on 35- or 60-mm culture dishes (Corning Glass Works, Corning, N.Y., USA) that were coated with rat tail collagen (500 μg dried tendon/ml in 0.9% acetic acid) and incubated in a humidified 5% CO_2_ incubator at 37°C.

### Treatment with EGTA and Ca^2+^ repletion assay

For the Ca^2+^ depletion experiment, the HPAC cells were treated with a calcium chelator, namely 2.5 mM EGTA, for 30 min and then cultured in medium without EGTA. For the calcium repletion assay, confluent HPAC cells were cultured in a low-Ca^2+^ medium (20 μM Ca^2+^) with 1% FBS overnight and then the medium was replaced by a normal medium containing 1.8 mM Ca^2+^.

### RNA interference and transfection

For RNA interference studies, small interfering RNAs (siRNAs) duplex-targeting the mRNA sequences of human tricellulin were purchased from Invitrogen. A scrambled siRNA sequence (BLOCK-iT Alexa Fluor fluorescent, Invitrogen) was employed as control siRNA. At 1 day before transfection, the HPAC cells were plated in medium without antibiotics so that they would be half-confluent at the time of transfection. The cells were transfected with 100 nM siRNAs by using Lipofectamine RNAiMAX (Invitrogen) as a carrier according to the manufacturer’s instructions.

### Western blot analysis

For Western blots of total cell lysates, the dishes were washed with phosphate-buffered saline (PBS) and 400 μl sample buffer (1 mM NaHCO_3_, 2 mM phenylmethylsulfonylfluoride) was added to the 60-mm culture dishes. The cells were scraped off, collected in microcentrifuge tubes and then sonicated for 10 s. The protein concentrations of samples were determined by using a BCA Protein Assay Reagent Kit (Pierce Chemical, Rockford, Ill., USA). Aliquots of 15 μg protein/lane for each sample were separated by electrophoresis in 4%/20% SDS polyacrylamide gels (Cosmo Bio, Tokyo, Japan). After electrophoretic transfer to nitrocellulose membranes (Immobilon; Millipore, Billerica, Mass., USA), the membranes were saturated with blocking buffer (Tris-buffered saline [TBS] with 0.1% Tween 20, 4% skim milk) for 30 min at room temperature and incubated with anti-tricellulin, anti-occludin, anti-claudin-1, anti-claudin-4, anti-claudin-7, anti-phosphothreonine, or anti-actin antibodies (1:1000) for 1 h at room temperature. The membranes were incubated with horseradish-peroxidase-conjugated anti-rabbit or mouse IgG (Dako, Copenhagen, Denmark) at room temperature for 1 h. Immunoreactive bands were detected by using an ECL Western blotting analysis system (GE Healthcare, Little Chalfont, UK).

### Triton-X-100-soluble and -insoluble fractions

The dishes were washed with PBS twice, lysed in Triton X-100 buffer (1% Triton X-100, 100 mM NaCl, 10 mM HEPES, pH 7.6, 2 mM EDTA, 1 mM phenylmethane sulfonyl fluoride [PMSF], 4 mM sodium orthovanadate, 40 mM sodium fluoride). The lysates were centrifuged at 15,000*g* for 20 min at 4°C. The soluble supernatant was collected and this fraction was defined as the Triton-X-100-soluble fraction. The pellet was solubilized in Triton X-100 buffer containing 1% SDS by using an ultrasonic disintegrator and cleared by centrifugation at 15,000*g* for 5 min at 4°C; this fraction was defined as the Triton-X-100-insoluble fraction. After the protein concentration of each sample had been quantified by using a BCA protein assay reagent kit (Pierce Chemical), the fractions were subjected to Western blot analysis with anti-tricellulin and anti-actin antibodies.

### Alkaline phosphatase treatment

Whole cell lysates and the Triton-X-100-insoluble fraction were incubated with 20 U calf intestine alkaline phosphatase (Takara Shuzo, Ohtsu, Japan) for 1 h at 37°C with occasional mixing and then subjected to Western blot analysis with anti-tricellulin and anti-actin antibodies.

### Immunoprecipitation

The dishes were washed with PBS twice and 300 μl NP-40 lysis buffer (50 mM Tris–HCl, 2% NP-40, 0.25 mM Na-deoxycholate, 150 mM NaCl, 2 mM EGTA, 0.1 mM Na_3_VO_4_, 10 mM NaF, 2 mM PMSF) was added to the 60-mm dishes. The cells were scraped off, collected in microcentrifuge tubes and then sonicated for 10 s. Cell lysates were incubated with protein A-Sepharose CL-4B (Pharmacia LKB Biotechnology, Uppsala, Sweden) for 1 h at 4°C and then clarified by centrifugation at 15,000*g* for 10 min. The supernatants were incubated with the polyclonal anti-tricellulin antibody bound to protein A-Sepharose CL-4B overnight at 4°C. After incubation, immunoprecipitates were washed extensively with the same lysis buffer and subjected to Western blot analysis with anti-tricellulin and anti-phosphothreonine antibodies.

### RNA isolation, reverse transcription polymerase chain reaction analysis and real-time polymerase chain reaction analysis

Total RNA was extracted and purified by using TRIzol (Invitrogen); 1 μg total RNA was reverse-transcribed into cDNA by using a mixture of oligo (dT) and Superscript II reverse transcriptase according to the manufacturer’s recommendations (Invitrogen). Synthesis of each cDNA was performed in a total volume of 20 μl for 50 min at 42°C and terminated by incubation for 15 min at 70°C. The polymerase chain reaction (PCR) was performed in a 20-μl total mixture containing 10 pmol primer pairs, 1.0 μl of the 20-μl total reverse transcription (RT) product, PCR buffer, dNTPs and *Taq* DNA polymerase according to the manufacturer’s recommendations (Takara, Kyoto, Japan). Amplifications were carried out for 25–35 cycles depending on the PCR primer pair with cycle times of 15 s at 96°C, 30 s at 55°C and 60 s at 72°C. The final elongation time was 7 min at 72°C. Of the total 20-μl PCR product, 7 μl was analyzed by 1% agarose gel electrophoresis with ethidium bromide staining and standardized by using a GeneRuler 100-bp DNA ladder (Fermentas, Ontario, Canada).

Real-time PCR detection was performed by using a TaqMan Gene Expression Assay kit with a StepOnePlus real-time PCR system (Applied Biosystems, Foster City, Calif., USA). The amount of 18S ribosomal RNA (Hs99999901) in each sample was used to standardize the quantity of the mRNA of tricellulin (Hs00930631). The relative mRNA expression levels between the control and treated samples were calculated by the difference of the threshold cycle (comparative C_T_ [∆C_T_] method) and presented as the average of triplicate experiments with a 95% confidence interval.

### Immunostaining

The cells were grown on 35-mm glass-base dishes (Iwaki, Chiba, Japan) coated with rat tail collagen and incubated with 10% FBS. They were then fixed with cold acetone and ethanol (1:1) at −20°C for 10 min. After being rinsed in PBS, they were incubated with polyclonal anti-tricellulin or monoclonal anti-occludin (1:100) at room temperature for 1 h and then with Alexa 488 (green)-conjugated anti-rabbit IgG and Alexa 594 (red)-conjugated anti-mouse IgG antibodies (1:200) at room temperature for 1 h. Some cells were double-stained by using polyclonal anti-tricellulin and monoclonal anti-occludin and were visualized with Alexa 488 (green)-conjugated anti-rabbit IgG and Alexa 594 (red)-conjugated anti-mouse IgG antibodies. Cell nuclei were counterstained with 4,6-diamidino-2-phenylindole (DAPI; Sigma-Aldrich). The specimens were examined by using an epifluorescence microscope (Olympus, Tokyo, Japan) and a confocal laser scanning microscope (LSM510; Carl Zeiss, Jena, Germany).

### Immuno-transmission electron microscopic analysis

HPAC cells grown to confluence on 60-mm tissue culture dishes were scraped from the dishes and collected in microcentrifuge tubes for immuno-transmission electron microscopic (immuno-TEM) analysis. After fixation with 4% paraformaldehyde and 0.1% glutaraldehyde in PBS for 20 min, the cells were incubated with 5% normal donkey serum in PBS for 15 min at room temperature to block nonspecific reactions. Then, they were incubated with the anti-tricellulin antibody (1:100) overnight at 4°C. After incubation, the cells were washed five times with PBS with Triton X-100 for 5 min each time and incubated with 12-nm colloidal-gold-conjugated anti-rabbit IgG (1:50). After five washes with PBS for 5 min each time, the specimens were fixed with 1.0% glutaraldehyde in PBS for 60 min at 4°C. The specimens were then rinsed with distilled water and incubated with 0.05 M HEPES solution for 30 min at room temperature. Following incubation, they were enhanced with a silver enhancement kit (Jackson Immuno Research Laboratories). The specimens were rinsed with distilled water, fixed with 2.5% glutaraldehyde in PBS for 90 min at room temperature, dehydrated through a graded ethanol series, embedded in epoxy resin and cut into ultrathin sections with a Sorvall MT6000 ultramicrotome (DEDUPOMT, New Castle, Del., USA). The ultrathin sections were stained with uranyl acetate and lead citrate and examined with a transmission electron microscope (H7500; Hitachi, Tokyo, Japan).

### Measurement of transepithelial electrical resistance

The cells were cultured to confluence on the inner chambers of 12-mm Transwells with 0.4-μm pore-size filters (Corning Life Science). Transepithelial electrical resistance (TER) was measured by using an EVOM voltmeter with an ENDOHM-12 (World Precision Instruments, Sarasota, Fla., USA) on a heating plate (Fine, Tokyo, Japan) adjusted to 37°C. The values are expressed in standard units of ohms per square centimeter and presented as means ± SD of triplicate experiments. For calculation, the resistance of blank filters was subtracted from that of filters covered with cells.

### Diffusion of BODIPY-sphingomyelin

For measurement of the tight-junctional fence function, we used the diffusion of BODIPY-sphingomyelin (Balda et al. 1996) with some modification (Kojima et al. 2008). Sphingomyelin/bovine serum albumin (BSA) complexes (5 nM) were prepared in P buffer (10 nM HEPES, pH 7.4, 1 mM sodium pyruvate, 10 mM glucose, 3 mM CaCl_2_, 145 mM NaCl) by using BODIPY-FL-sphingomyelin (Molecular Probes) and defatted BSA. Cells plated on glass-bottom microwell plates (Mat Tek, Ashland, Mass., USA) were loaded with the BODIPY-sphingomyelin/BSA complex for 1 min on ice, after which they were rinsed with cold DMEM and mounted in DMEM on a glass slide. The samples were analyzed by confocal laser scanning microscopy (LSM510; Carl Zeiss).

### Data analysis

Signals were quantified by using Scion Image Beta 4.02 Win (Scion, Frederick, Mass., USA). Each set of results shown is representative of at least three separate experiments. Results are given as means ± SE. Differences between groups were tested by analysis of variance followed by a post hoc test and an unpaired two-tailed Student’s *t*-test and considered to be significant when *P*<0.05.

## Results

### Effects of intracellular Ca^2+^ on expression and localization of tricellulin in HPAC cells

**Fig. 1:**
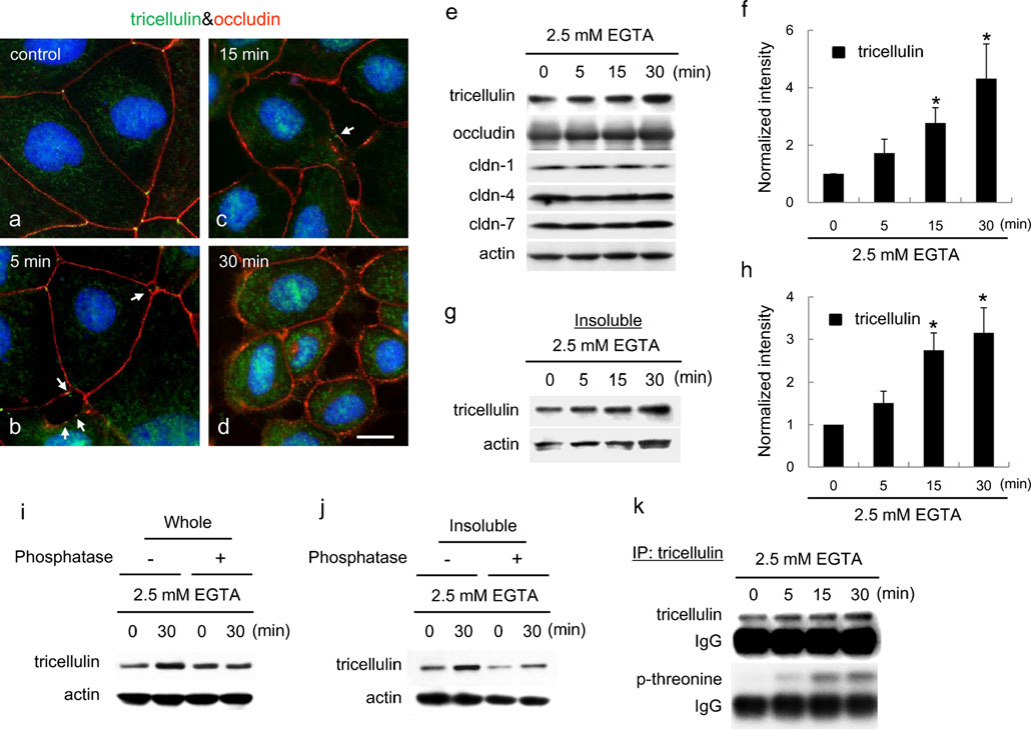
Expression and distribution of tricellulin in the human pancreatic cancer cell line (HPAC) after treatment with EGTA. **a–d** Immunostaining images obtained by using double-staining for tricellulin (*green*) and occludin (*red*) in HPAC cells after treatment with 2.5 mM EGTA (*arrows* gaps). Nuclei are shown in *blue* (DAPI staining) *Bar* 20 μm. **e** Western blotting for tricellulin, occludin, claudin (*cldn*)-1, -4 and -7 and actin in whole cell lysates of HPAC cells after treatment with 2.5 mM EGTA. Expression levels of tricellulin are shown in **f** in a *bar graph* (*n*=4,*P<0.05 versus 0 h). **g** Western blotting for tricellulin in the Triton-X-100-insoluble fraction (*Insoluble*) of HPAC cells after treatment with 2.5 mM EGTA. Expression levels of tricellulin are shown in **h** in a *bar graph* (*n*=4,*P<0.05 versus 0 h). **i**, **j** Western blotting for tricellulin in whole cell lysates (*Whole*) and in the Triton-X-100-insoluble fraction (*Insoluble*). After 2.5 mM EGTA treatment, HPAC cells were lysed and then treated with or without alkaline phosphatase, followed by detergent extraction. **k** Western blotting for phosphothreonine in immunoprecipitates (*IP*) by using an anti-tricellulin antibody in HPAC cells after treatment with 2.5 mM EGTA

Tricellulin was expressed at tricellular contacts in differentiated human pancreatic duct carcinoma cell line HPAC as we previously reported (Fig. 1a; Kojima et al. 2010). To investigate the effects of intracellular Ca^2+^ on the expression and localization of tricellulin in HPAC cells, the cells were treated with a calcium chelator, namely 2.5 mM EGTA.

When the membranes of tricellular contacts began to detach, gaps formed at these regions from 5 min after EGTA treatment but tricellulin was initially maintained at the membranes around the gap formation (Fig. 1b). At 30 min after treatment, tricellulin was not detected at the membranes and was observed in the cytoplasm (Fig. 1d). Occludin remained in membranes for 30 min during EGTA treatment. In Western blots of whole lysates, tricellulin was increased from 5 min after EGTA treatment and was significantly upregulated 2.6-fold and 4.3-fold compared with the control level at 15 and 30 min after treatment with EGTA, respectively, whereas occludin and claudin-1, -4 and -7 were unchanged (Fig. 1e, f). Triton-X-100-soluble and -insoluble fractions are operational definitions that have been used to define the localization of tight junction proteins biochemically (Nusrat et al. 2000). Because tricellulin was invariably found to be Triton-X-100-insoluble, only Triton-X-100-insoluble fractions were analyzed. Under the same exposure time as for Western blotting, the amount of tricellulin in the Triton-X-100-soluble fraction was small compared with that in the Triton X-100-insoluble fraction (Supplemental Fig. 2). From 5 min after treatment with 2.5 mM EGTA, tricellulin increased in the Triton-X-100-insoluble fractions (Fig. 1g, h). Treatment of whole cell lysates with alkaline phosphatase prevented the upregulation of tricellulin in whole lysates and Triton-X-100-insoluble fractions at 30 min after treatment with EGTA (Fig. 1i, j).

In immunoprecipitates examined by using the anti-tricellulin antibody, phosphothreonine was detected and increased from 5 min after EGTA treatment (Fig. 1k).

 In RT-PCR and real-time RT-PCR, no changes of mRNAs of tricellulin, occludin and claudin-1, -4, and -7 were observed after EGTA treatment (Supplemental Fig. 1a, b).

**Fig. 2:**
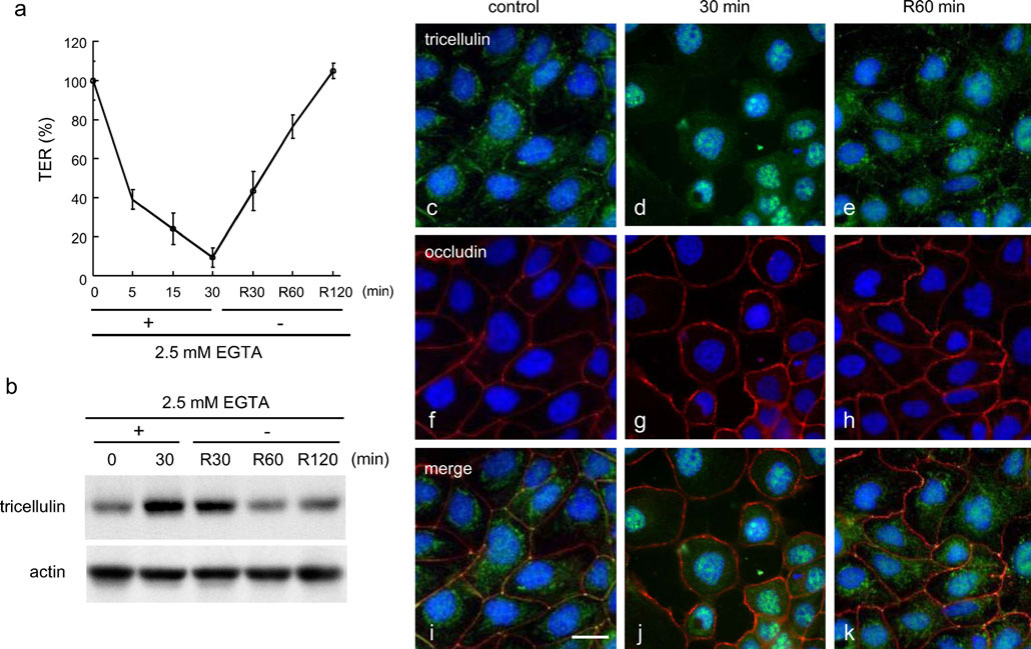
**a** Transepithelial electrical resistance (TER) values. **b** Western blotting for tricellulin. **c–k** Immunostaining of tricellulin and occludin in HPAC cells. After treatment with 2.5 mM EGTA for 30 min, HPAC cells were transferred to normal medium without EGTA (*R* recovery). *Bar* 20 μm

After treatment with 2.5 mM EGTA for 30 min, the cells were transferred to normal medium without EGTA. As shown in Fig. 2a, TER values indicated that the tight junction barrier gradually decreased until 30 min after EGTA treatment and then promptly recovered to normal levels after 120 min without EGTA treatment (Fig. 2a). The upregulation of tricellulin at 30 min after EGTA treatment in Western blots was completely inhibited after 60 min without EGTA treatment (Fig. 2b). In immunostaining, tricellulin, which had disappeared from the membranes at 30 min after EGTA treatment, was recruited into the membranes of tricellular contacts after 60 min without EGTA treatment (Fig. 2c–k). After 60 min without EGTA treatment (Fig. 2, R60 min), the localization of tricellulin showed heterogeneity. Tricellulin was observed at various positions including not only tricellular tight junctions but also bicellular tight junctions and cytoplasm.

### Changes of expression of tricellulin during Ca^2+^ repletion in HPAC cells

As shown in Fig. 2c–k, removal of EGTA resulted in a heterogeneous localization of tricellulin. Most tricellulin was immediately localized at tricellular tight junctions but some was still in the cytoplasm. Therefore, we decided to examine the behavior of tricellulin during the formation of tight junctions under various experimental conditions. To achieve homogeneous culture with Ca^2+^ starvation, HPAC cells were cultured in a low-Ca^2+^ medium (containing 20 μM Ca^2+^) overnight under confluent conditions. These Ca^2+^-starved cultures were transferred to normal Ca^2+^ medium (1.8 mM Ca^2+^) and the behavior of tricellulin during the formation of tight junctions was investigated.

**Fig. 3:**
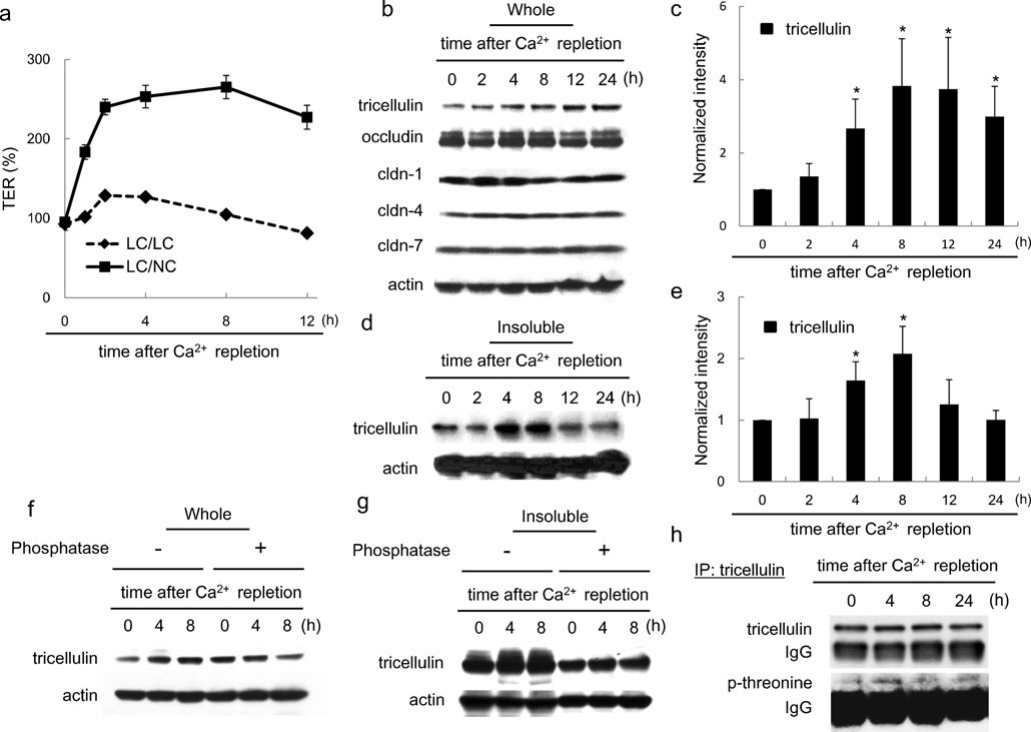
Changes of expression of tricellulin and barrier function during the Ca^2+^ repletion in HPAC cells. **a** TER values in HPAC cells during the Ca^2+^ repletion. HPAC cells were cultured in a low-Ca^2+^ medium (20 μM Ca^2+^; *LC*) overnight and then transferred to normal Ca^2+^ medium (*LC/NC*) or to fresh low-Ca^2+^ medium (*LC/LC*). **b** Western blotting for tricellulin, occludin and claudin (cldn) -1, -4 and -7 in whole cell lysates of HPAC cells (*Whole*) after Ca^2+^ repletion. Expression levels of tricellulin are shown in **c** in a *bar graph* (*n*=4, *P<0.05 versus 0 h). **d** Western blotting for tricellulin in the Triton-X-100-insoluble fraction (*Insoluble*) of HPAC cells after Ca^2+^ repletion. Expression levels of tricellulin are shown in **e** in a *bar graph* (*n*=3, *P<0.05 versus 0 h). **f**, **g** Western blotting for tricellulin in whole cell lysates (*Whole*) and in the Triton-X-100-insoluble fraction (*Insoluble*) of HPAC cells with or without alkaline phosphatase up until 8 h after Ca^2+^ repletion. **h** Western blotting for phosphothreonine after immunoprecipitation (*IP*) by using an anti-tricellulin antibody in HPAC cells after Ca^2+^ repletion

As shown in Fig. 3a, the TER value rapidly increased on Ca^2+^ repletion in a time-dependent manner and reached a plateau at 4 h after Ca^2+^ repletion. In Western blots of whole lysates, tricellulin increased from 2 h after Ca^2+^ repletion and was significantly upregulated from 4 h until 24 h, with a maximal level at 8 h (1.31-fold at 2 h; 2.71-fold at 4 h; 3.62-fold at 8 h; 3.47-fold at 12 h; 2.89-fold at 24 h), whereas no changes of occludin or claudin-1, -4 and -7 were observed (Fig. 3b, c). As shown in Fig. 3d, e, tricellulin in Triton-X-100-insoluble fractions reached a maximal level from 4 h to 8 h after Ca^2+^ repletion and then declined to the control level at 24 h. Under the same exposure time as for Western blotting, the amount of tricellulin in the Triton-X-100-soluble fraction was small compared with that in the Triton-X-100-insoluble fraction, as described above for EGTA treatment (Supplemental Fig. 3). Treatment with alkaline phosphatase prevented the upregulation of tricellulin in whole lysates and Triton-X-100-insoluble fractions from 4 h to 8 h after Ca^2+^ repletion (Fig. 3f, g). Notably, in Triton-X-100-insoluble fractions, an increasing width caused by an upward band shift became evident after 4 h and treatment with alkaline phosphatase significantly decreased the apparent molecular mass of the tricellulin band. In immunoprecipitation assays with the anti-tricellulin antibody, phosphothreonine was maximally increased at 4 h after Ca^2+^ repletion (Fig. 3h).

In RT-PCR and real-time PCR, no changes of mRNAs of tricellulin, occludin, or claudin-1, -4 and -7 were observed during Ca^2+^ repletion (Supplemental Fig. 1c, d).

### Changes of localization of tricellulin during Ca^2+^ repletion in HPAC cells

**Fig. 4:**
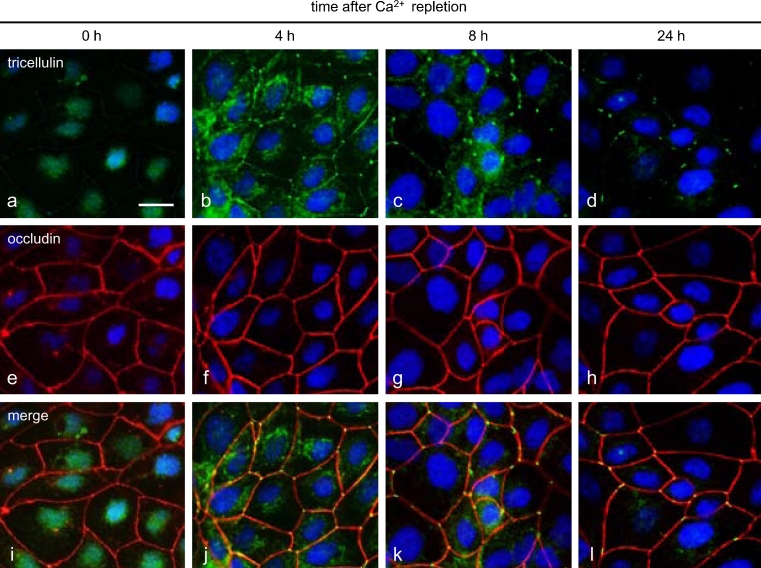
**a–l** Immunostaining images obtained by using double-staining for tricellulin and occludin in HPAC cells after Ca^2+^ repletion. *Bar* 20 μm

**Fig. 5:**
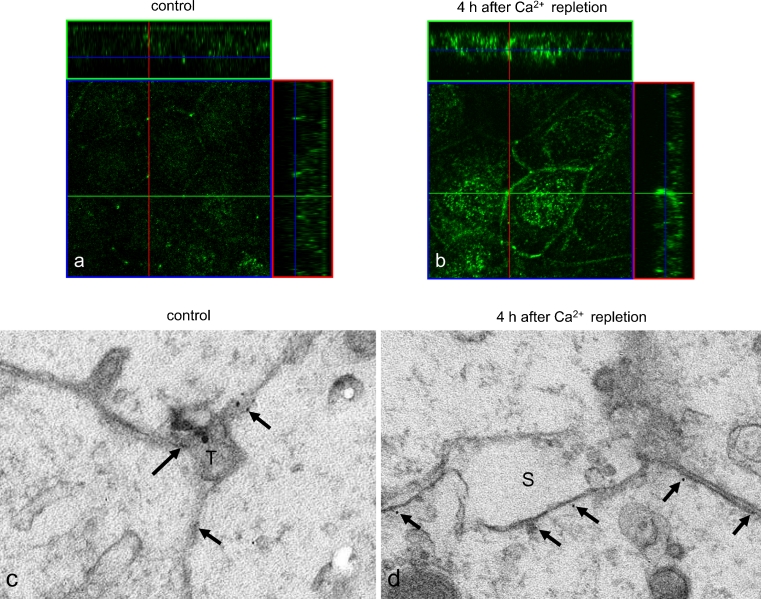
**a**, **b** Confocal laser scanning microscopic images of tricellulin in HPAC cells at 4 h after Ca^2+^ repletion. *Bar* 20 μm. **c**, **d** Immuno-transmission electron microscopic images of tricellulin in HPAC cells at 4 h after Ca^2+^ repletion (*T* tricellular contact, *S* space, *arrows* immunogold particles directed against tricellulin). *Bar* 50 nm

The localization of tricellulin during the formation of tight junctions over the course of Ca^2+^ repletion was examined by immunostaining and immuno-TEM analysis. During low-Ca^2+^ treatment, no tricellulin was detected in any cytoplasmic membranes (Fig. 4). At 4 h after Ca^2+^ repletion, tricellulin immunoreactivity was observed not only at tricellular contacts but also in the cytoplasm and at bicellular borders. By 8 h after Ca^2+^ repletion, tricellulin immunoreactivity had decreased in the cytoplasm and at bicellular borders but was strongly detected at tricellular contacts. At 24 h after Ca^2+^ repletion, tricellulin was recruited to the membranes of tricellular contacts. In Z-sections obtained by confocal laser microscopy, tricellulin was observed not only at the apical-most regions but also at basolateral membranes at 4 h after Ca^2+^ repletion, whereas in control cells, it was observed at the apical-most regions of tricellular contacts (Fig. 5a, b).

In the control, some immunogold particles directed against tricellulin were observed on the membranes around the closed tricellular contact by immuno-TEM analysis (Fig. 5c). At 4 h after Ca^2+^ repletion, the immunogold particles were observed along the membranes, around small spaces and at bicellular borders (Fig. 5d).

### Effects on barrier and fence functions during Ca^2+^ repletion by knockdown of tricellulin in HPAC cells

To investigate whether knockdown of tricellulin by siRNAs affected the barrier and fence functions of tight junctions during Ca^2+^ repletion, we examined TER for barrier function assays and performed diffusion assays with BODIPY-sphingomyelin for fence function assay.

**Fig. 6:**
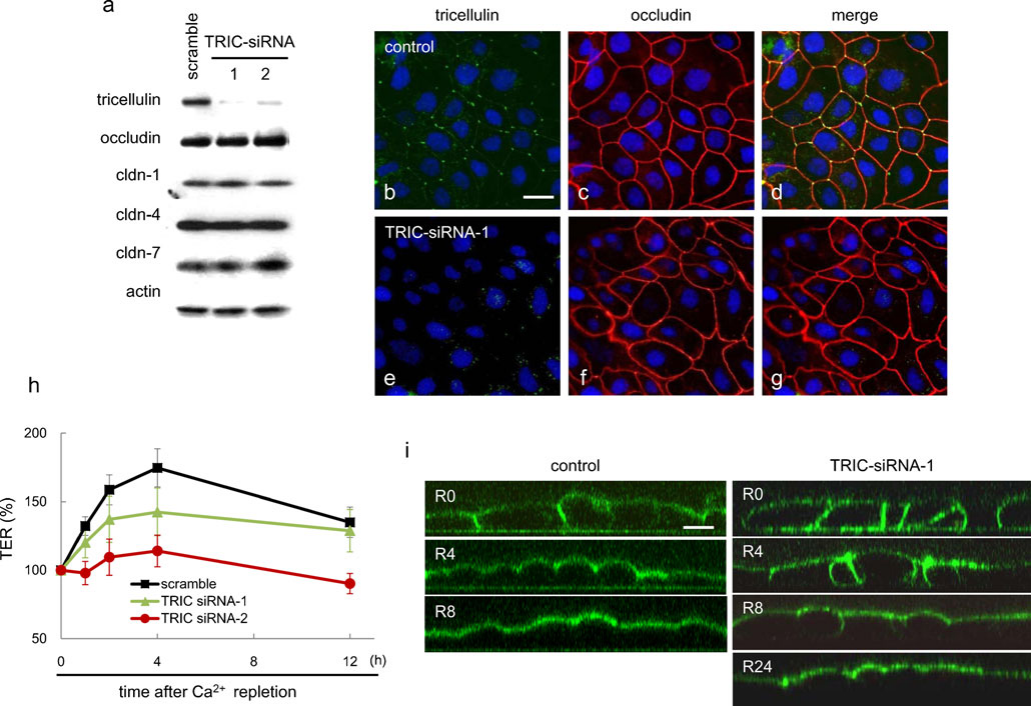
Effects of small interfering RNA (*siRNA*) for tricellulin (*TRIC*) on the barrier and fence functions in HPAC cells after a Ca^2+^ repletion. **a** Western blotting for tricellulin, occludin and claudin (*cldn*) -1, -4, and -7 in whole cell lysates of HPAC cells treated with siRNAs for tricellulin and the negative scrambled control. **b–g** Immunostaining images obtained by using double-staining for tricellulin and occludin in HPAC cells treated with siRNA-1 for TRIC. *Bar* 20 μm. **h** TER values in HPAC cells pretreated with siRNAs for tricellulin after Ca^2+^ repletion. **i** Fence function as examined by diffusion of labeled BODIPY-sphingomyelin through HPAC cell layer pretreated with siRNA for tricellulin before Ca^2+^ repletion (*R* recovery)

At 48 h after transfection with two sets of 100 nM siRNAs for tricellulin, tricellulin was decreased compared with the control in Western blots, whereas no changes in occludin or claudin-1, -4 and -7 were observed (Fig. 6a). At 48 h after transfection with 100 nM siRNA-1 for tricellulin, tricellulin-positive spots disappeared at the tricellular contacts, whereas occludin was expressed at the cell borders as in the control (Fig. 6b–g). In RT-PCR, tricellulin mRNA decreased compared with the control, whereas no changes of occludin or claudin-1, -4 and -7 were observed (Supplemental Fig. 1e).

The increase of TER values until 4 h after Ca^2+^ repletion was inhibited by the two sets of 100 nM siRNAs for tricellulin compared with the control (Fig. 6h). BODIPY-sphingomyelin was effectively retained at the apical domain of control cells. At 0 h after Ca^2+^ repletion, the BODIPY-sphingomyelin diffused through the tight junctions, being strongly labeled at the basolateral and basal surfaces and appearing to penetrate the cells, indicating that the fence function of tight junctions was reduced. At 4 h after Ca^2+^ repletion, diffusion of the probe was markedly decreased compared with that of 0 h. The probe was effectively retained in the apical domain at 8 h after Ca^2+^ repletion. In cells transfected with 100 nM siRNA-1 for tricellulin, diffusion of the probe was in part observed at the basolateral surfaces at 4 h and 8 h after Ca^2+^ repletion.

## Discussion

To elucidate the roles of tricellulin in the establishment of tight junctions, we examined the behavior of endogenous tricellulin during the process of the disruption and formation of tight junctions achieved by modulating extracellular Ca^2+^ conditions in the polarized human pancreatic cell line HPAC. Our results provide evidence showing that the dynamic changes of the distribution and phosphorylation of tricellulin in the course of the destruction and formation of tight junctions are probably linked to the barrier and fence functions of tight junctions.

Ca^2+^ depletion caused by EGTA treatment induced the disruption of tight junctions and displaced the tricellulin from tricellular contacts to bicellular junctions and finally to the cytoplasm; this was correlated with a significant reduction in TER (Figs. 1, 2). These alterations of tricellulin localization and TER were promptly reversed by the removal of EGTA. Under the same conditions, no significant change was observed in the localization of occludin. Ca^2+^ repletion after Ca^2+^ starvation induced tight junction formation and altered the localization of tricellulin from the cytoplasm to the basolateral membranes of bicellular junctions and finally to the apical sites of tricellular contacts (Fig. 4).

In the course of the Ca^2+^-dependent alterations of tricellulin localization, the band intensities and widths of tricellulin in Western blots transiently increased in both the whole lysate and the Triton-X-100-insoluble fraction without changes in its mRNA level (Figs. 1, 3; Supplemental Fig. 1). A possible cause for the increased intensity and width was the phosphorylation of the tricellulin protein, as previously described for occludin (Antonetti et al. 1999; Farshori and Kachar 1999; Andreeva et al. 2001). Consistently, when the intensity or width increased as a result of the upward band shift of tricellulin, protease-free alkaline phosphatase treatment reduced the band intensity or width, suggesting that the tricellulin was phosphorylated (Figs. 1, 3). Indeed, immunoprecipitation with the anti-tricellulin antibody confirmed that phosphothreonine considerably increased with the increasing band intensity of tricellulin in both the whole lysate and Triton-X-100-insoluble fraction (Figs. 1, 3), indicating that phosphorylated tricellulin was resistant to detergent extraction, similar to occludin (Sakakibara et al. 1997; Wong 1997). Tricellulin was transiently phosphorylated on threonine residue(s) when its localization changed, suggesting that phosphorylation might be closely associated with tricellulin displacement, i.e., internalization, during the disruption and formation of tight junctions. However, these results cannot completely exclude the possibility that EGTA treatment had inhibited the degradation of tricellulin and resulted in the increase of its band intensity, even though the band intensities of claudins and occludin were not altered under the same conditions.

The phosphorylation of tight junction proteins appears to play a central role in the regulation of tight junction assembly and function. The phosphorylation of occludin has been well examined in association with its localization and function (Sakakibara et al. 1997; Wong 1997; Seth et al. 2007; Murakami et al. 2009). As a result of experiments on confluent MDCK cells and Ca^2+^ repletion, the redistribution and phosphorylation of occludin during the opening and resealing of tight junctions has become well-known (Farshori and Kachar 1999). Like occludin, tricellulin appears to be phosphorylated (Ikenouchi et al. 2005). We have previously reported that threonine phosphorylation of tricellulin in HPAC cells is induced by treatment with the JNK activator anisomycin (Kojima et al. 2010). Whether these threonine residues are phosphorylated sequentially or randomly in each molecule or whether the phosphorylation of some residues is functionally more important than that of others remains elusive. The phosphorylation is sensitive to extracellular Ca^2+^ conditions, as for occludin but not for claudins (Farshori and Kachar 1999; Kubota et al. 1999). However, the specific kinases responsible for tricellulin phosphorylation are unclear, whereas occludin is phosphorylated by various kinases, including protein kinase C, protein kinase A, mitogen-activated protein kinases, phosphatidylinositol 3-kinase and the Ser/Thr kinases, namely casein kinase 1 and casein kinase 2 (Dörfel et al. 2009). Since the C-terminus cytoplasmic sequence of tricellulin is highly similar to that of occludin, tricellulin might also bind to various kinases. Tricellulin is also thought to be present along the lateral membrane at tricellular tight junctions (Raleigh et al. 2010; Mariano et al. 2011). In the present study, at 4 h after Ca^2+^ repletion, tricellulin induced in the Triton-X-100-insoluble fraction and phosphorylated on threonine residues was localized not only at tricellular contacts but also in cytoplasm and at bicellular borders as shown by immunofluorescence microscopy and immuno-TEM analysis (Fig. 5). In Z-sections obtained by confocal laser microscopy, tricellulin was observed at the apical-most regions and basolateral membranes of tricellular contacts at 4 h after Ca^2+^ repletion (Fig. 5). The way in which tricellulin is trafficked to the tricellular contacts remains unclear, as is the manner in which the bicellular tight junction proteins occludin and claudins are trafficked to the tight junction regions. These results suggest that tricellulin is trafficked to the membranes of the bicellular borders and the basolateral membranes and then to the apical-most regions at the tricellular contacts during the formation of tight junctions. The phosphorylation of tricellulin might play an important role in these processes, as described above.

Although tricellulin expression is closely associated with the barrier function (Ikenouchi et al. 2005; Krug et al. 2009), the relationship between the phosphorylation of tricellulin and the barrier function is unclear. Furthermore, we do not know whether tricellulin affects the fence function. In the present study, when the dynamic changes of the distribution and phosphorylation of tricellulin were observed by using Ca^2+^ repletion, the knockdown of tricellulin by siRNAs delayed the recovery of both the barrier and fence functions after Ca^2+^ repletion (Fig. 6). These findings indicate that the expression of tricellulin might be closely associated with the barrier and fence functions during the formation of tight junctions.

In conclusion, the dynamic behavior of tricellulin, including phosphorylation, has been observed during the destruction and formation of tight junctions under various extracellular calcium conditions and is closely associated with both the barrier and fence functions of tight junctions. Tricellulin directly binds to marvelD3 and zonula occludens protein 1 (Riazuddin et al. 2006; Raleigh et al. 2010; Westphal et al. 2010) and forms homomeric complexes and heteromeric complexes with occludin (Westphal et al. 2010). More recently, Masuda and colleagues (2011) have reported that the cytoplasmic domain of LSR is responsible for the recruitment of tricellulin. The phosphorylation and dephosphorylation of tricellulin might play a role in its interaction with other tight junction proteins including LSR at tricellular tight junctions. Whether ubiquitylation is involved in the turnover and trafficking of tricellulin, as for occludin and claudins (Takahashi et al. 2009; Traweger et al. 2002), remains unclear. The mechanism and role of tricellulin phosphorylation need to be elucidated in future studies.

## Electronic supplementary material

Below is the link to the electronic supplementary material.

ESM 1 (PDF 232 kb)
