# Supplementary material for: Behavior of tricellulin during destruction and formation of tight junctions under various extracellular calcium conditions
Source: Cell Tissue Res. 2012 Oct 17;351(1):73–84. doi: 10.1007/s00441-012-1512-7 (PMC3536962; doi:10.1007/s00441-012-1512-7)
Supplement: Supplementary file 1 — (PDF 232 kb) [file 441_2012_1512_MOESM1_ESM.pdf]

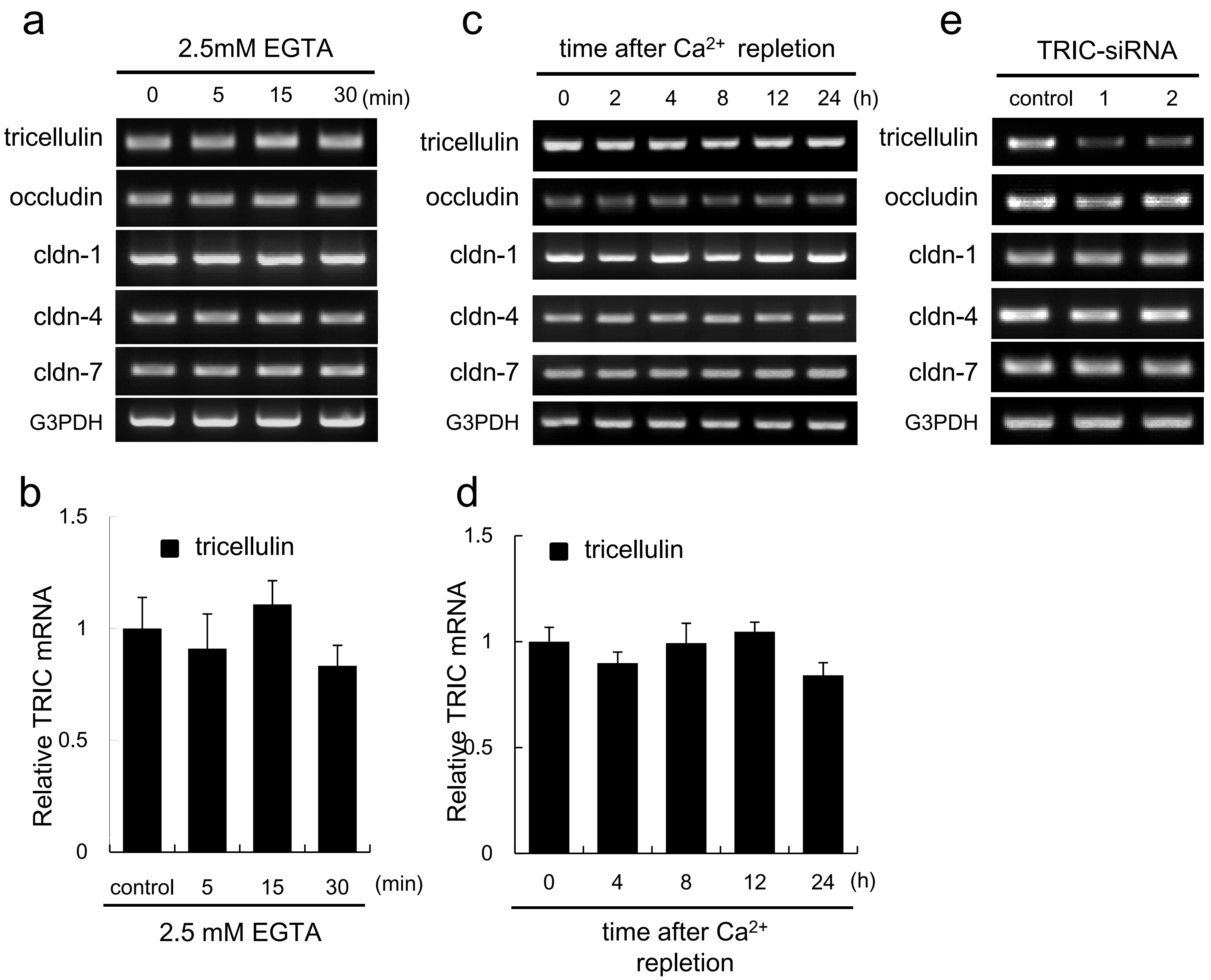

Supplemental Fig. 1 RT-PCR for tricellulin, occludin, claudin (cldn) -1, -4, and -7 in HPAC cells after treatment with 2.5 mM EGTA (a), after Ca^{2+} repletion (c) and after treatment with siRNA of tricellulin (e). Expression levels of tricellulin are shown in a and c as bar graphs of b and d.

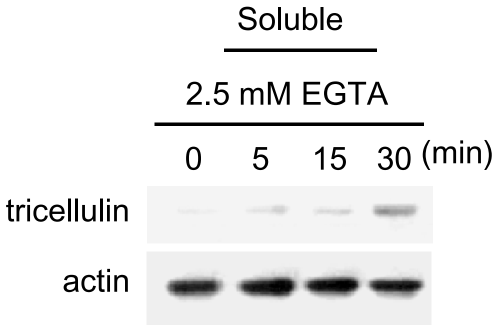

Supplemental Fig. 2 Changes of expression of tricellulin in HPAC cells after treatment with EGTA. Western blotting for tricellulin in the Triton X-100-soluble fractions of HPAC cells after treatment with 2.5 mM EGTA

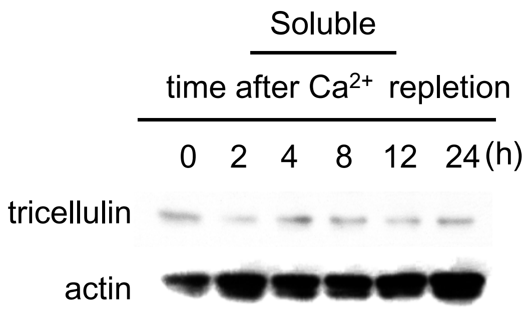

Supplemental Fig. 3 Changes of expression of tricellulin during Ca^{2+} repletion in HPAC cells. Western blotting for tricellulin in the Triton X-100-soluble fractions after Ca^{2+} repletion.
